# Synthesis of rigidified flavin–guanidinium ion conjugates and investigation of their photocatalytic properties

**DOI:** 10.3762/bjoc.5.26

**Published:** 2009-05-28

**Authors:** Harald Schmaderer, Mouchumi Bhuyan, Burkhard König

**Affiliations:** 1 Institute of Organic Chemisty, University of Regensburg, Universitätsstr. 31, D-93040 Regensburg, Germany

**Keywords:** flavin, guanidine, Kemp’s acid, photocatalysis, template

## Abstract

Flavin chromophores can mediate redox reactions upon irradiation by blue light. In an attempt to increase their catalytic efficacy, flavin derivatives bearing a guanidinium ion as oxoanion binding site were prepared. Chromophore and substrate binding site are linked by a rigid Kemp’s acid structure. The molecular structure of the new flavins was confirmed by an X-ray structure analysis and their photocatalytic activity was investigated in benzyl ester cleavage, nitroarene reduction and a Diels–Alder reaction. The modified flavins photocatalyze the reactions, but the introduced substrate binding site does not enhance their performance.

## Introduction

**Scheme 1:**
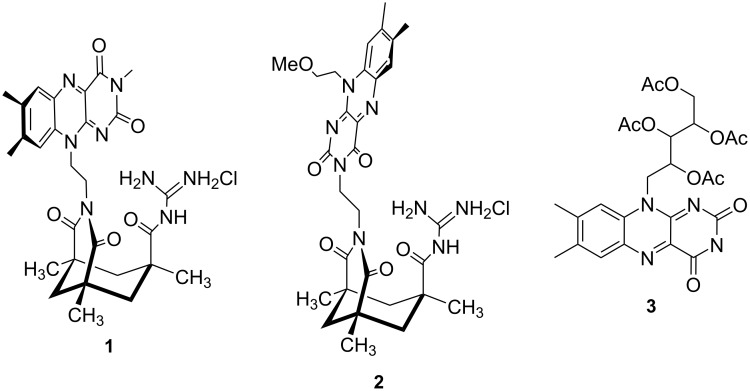
Flavin–guanidinium ion conjugates **1** and **2** and tetraacetyl riboflavin (**3**).

Flavins are redox-active chromophores [1–6] and represent one of the most abundant classes of natural enzyme co-factors [7–9]. Recently, the photo redox properties of flavins have been used to catalyze chemical reactions [10–30]. A general drawback of photochemical processes in homogeneous solution is the limited preorganization of the reactants and the chromophore, which may lead to low selectivities and slow conversions in diffusion controlled reactions. To overcome this problem, Kemp’s acid [31] derivatives have been used as sterically defined templates enhancing the efficiency and selectivity of photoreactions [32–43]. Flavins with geometrically defined substrate binding sites have not been reported so far and we expected that the close vicinity of substrate and flavin should enhance the rate of photoinduced electron transfer processes, which strongly depend on distance [44]. We present here the synthesis of geometrically defined flavin-guanidinium ion conjugates based on a Kemp’s acid skeleton (Scheme 1). The guanidinium moiety should serve as a hydrogen bonding site for oxoanions or carbonyl groups [45–49]. The structure of the new flavins was determined in solid state and in solution and their photocatalytic properties were tested.

## Results and Discussion

### Synthesis

**Scheme 2:**
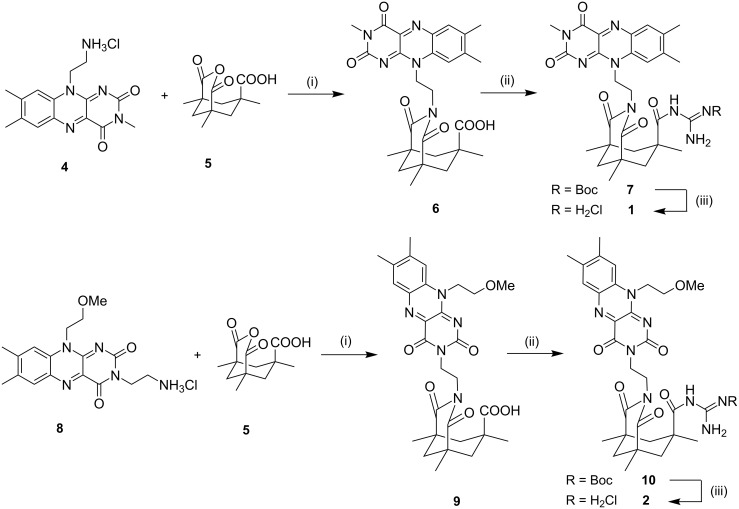
Synthesis of flavins **1** and **2**. Conditions: (i) DMAP, H_2_O, Δ, 20 h, 71–78%, (ii) HOBt, EDC, NEt(*i*Pr)_2_, mono-Boc guanidine, CH_2_Cl_2_, rt, 20 h, 58–82%, (iii) HCl/Et_2_O, CH_2_Cl_2_/CHCl_3_, rt, 24 h, 83–90%.

The synthesis of the potential photocatalysts **1** and **2**, consisting of the flavin chromophore, the guanidinium substrate binding site and a Kemp’s acid derived rigid linker, starts from Kemp’s acid anhydride (**5**) [50–52]. The anhydride **5** was allowed to react with previously prepared flavins **4** and **8** [21] in the presence of DMAP as catalyst. The amide formation of the carboxyl group with Boc-protected guanidine was achieved using standard peptide coupling conditions. Boc-deprotection with hydrogen chloride in diethyl ether yielded the guanidinium chloride salts **1** and **2** (Scheme 2). The guanidinium salts are soluble in water and methanol, but also in chloroform and acetonitrile.

### Structural investigations

**Figure 1:**
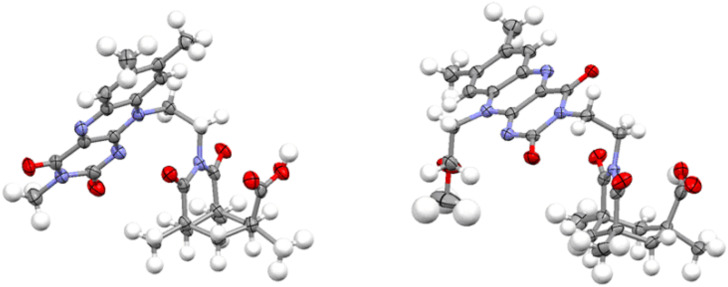
X-ray crystal structures of the flavin-Kemp’s acids **6** (left) and **9** (right).

The structure of compounds **1**, **2**, **6**, and **9** was examined in the solid state and in solution. Figure 1 shows the X-ray crystal structures of **6** and **9**. The planar flavin chromophore is turned outward relative to the Kemp’s acid. Intermolecular π-π-interactions between the flavin heteroarenes are observed.

**Figure 2:**
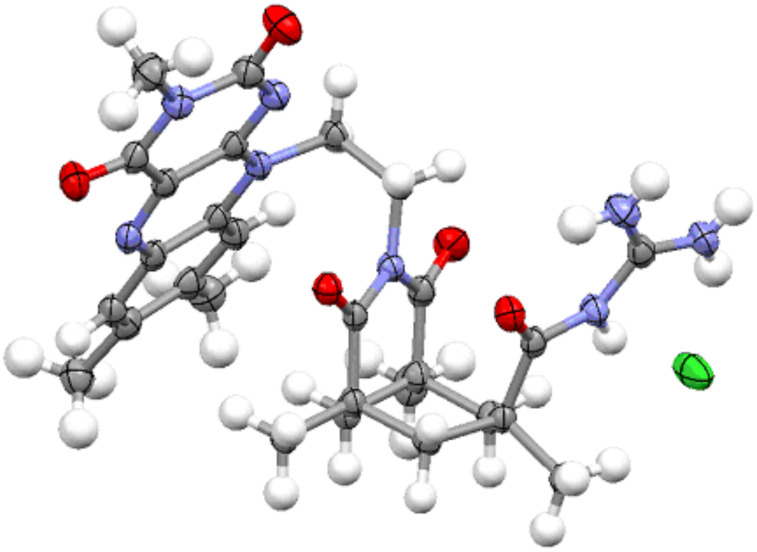
Structure of compound **1** in the solid state.

The structure of compound **1** in the solid state (Figure 2) shows an almost identical orientation of the flavin group to that of the acid **6**. The acyl guanidinium ion group is almost planar and in a parallel orientation relative to the Kemp’s acid imide group.

2-D NMR spectra of compounds **1** and **2** revealed several NOE contacts, but the flexibility of the molecule did not allow the determination of preferred conformations.

**Figure 3:**
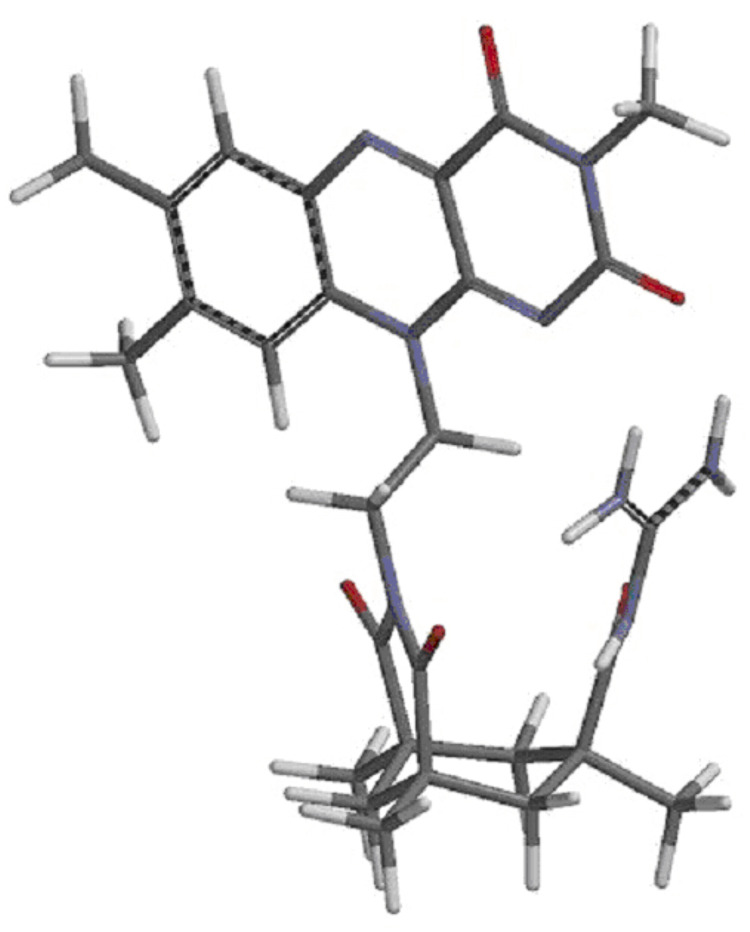
Calculated lowest energy conformation of **1** in the gas phase (AM1, Spartan program package).

The most stable conformer of compound **1** in the gas phase was determined by computational methods (semi-empirical AM1, Spartan program package, Figure 3, see also Supporting Information File 1) [53]. In this structure the flavin is turned towards the guanidinium ion forming a hydrogen bond between the flavin carbonyl oxygen atom and the guanidinium moiety (distance ~2.1 Å). However, simple gas phase calculations overestimate the effect of hydrogen bonds [54–58] and in solution the flavin chromophore is expected to rotate freely around the C–C single bonds of the ethane linker.

### Photocatalytic reactions

**Scheme 3:**
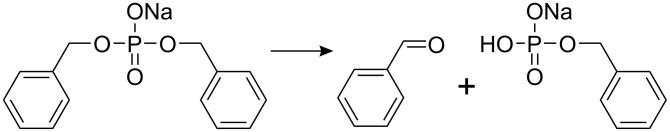
Oxidative photocleavage of dibenzyl phosphate.

Compounds **1** and **2** were tested as photocatalysts in three different reactions and their performance was compared to tetraacetyl riboflavin **3** or compound **8**. Dibenzyl phosphate esters are oxidatively cleaved by blue light irradiation (440 nm) in the presence of compounds **1** and **2** (Scheme 3). The acceleration of the reaction in acetonitrile by **1** and **2**, bearing a guanidinium ion binding site with phosphate affinity, is significantly larger (Table 1, entries 1+2) in comparison to the ammonium salt **8** (entry 3). In water, however, the accelerating effect is not observed (entries 5–8). The presence of the photocatalyst is essential in all cases, as the non-catalyzed hydrolysis is slow under the reaction conditions (<5% conversion).

**Table 1:** Oxidative photocleavage of dibenzyl phosphate.

Entry	Catalyst	Solvent	t (h)	Conversion (%)

1^a^	**1**	MeCN-*d_3_*	4	53
2^a^	**2**	MeCN-*d_3_*	4	58
3^a^	**8**	MeCN-*d_3_*	4	12
4^a^	–	MeCN-*d_3_*	4	<5

5	**1**	D_2_O	2	44
6	**2**	D_2_O	2	15
7	**8**	D_2_O	2	50
8	–	D_2_O	2	<5

Conditions: V = 1 mL, dibenzyl phosphate 10^−2^ M, catalyst 20 mol%, 40 °C, LED (440 nm). ^a^Dibenzyl phosphate ester was neutralized previous to the reaction.

**Scheme 4:**
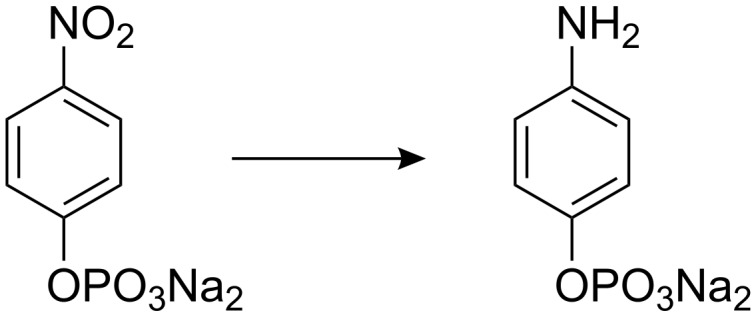
Photoreduction of 4-nitrophenyl phosphate.

In the presence of sacrificial electron donor substrates, such as aliphatic amines, flavins can photoreduce nitro arenes to anilines under blue light irradiation (Scheme 4). 4-Nitrophenyl phosphate was used as a substrate for photoreduction in water and in acetonitrile. The results summarized in Table 2 show that 10 mol% of flavin **2**, the same amount of tetraacetyl riboflavin (**3**) or compound **8** catalyze the photoreaction equally well. The guanidinium ion binding site of **1** and **2** does not lead to a more effective conversion.

The intermolecular interaction of the guanidinium ion binding site of compound **2** with phosphate ester anions and dianions was probed by UV/vis and emission spectroscopy in acetonitrile and buffered aqueous solution. The emission intensity of the chromophore of **2** decreased slightly in the presence of the anions in acetonitrile indicating a weak interaction. In aqueous solution the presence of the anions did not induce significant changes of the emission properties suggesting affinity constants smaller than 10^3^ L/mol.

**Table 2:** Results of nitrobenzene photoreduction.

Entry	Catalyst	Solvent	Conversion (%)

1	**1**	H_2_O	36
2	**2**	H_2_O	72
3	**2**	H_2_O^a^	73
4	**3**	H_2_O^a^	89
5	**8**	H_2_O	79

6^b^	**1^c^**	MeCN	15
7^b^	**2**	MeCN	55
8^b^	**3**	MeCN	81
9^b^	**8**	MeCN	59

Conditions: V = 5 mL, nitrobenzene 10^−2^ M, catalyst 10 mol%, N(CH_2_CH_2_OH)_3_ 10 equiv, t = 4 h, 40 °C, LED (440 nm), UV-lamp (370 nm). ^a^10% DMSO added to increase solubility. ^b^4-Nitrophenyl phosphate was neutralized previous to the reaction. ^c^The catalyst is barely soluble in MeCN, which explains the lower conversion in this case.

**Scheme 5:**
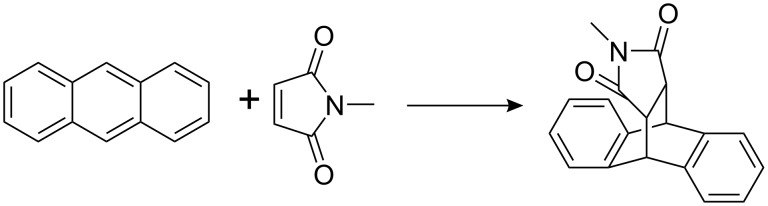
Photo Diels–Alder-reaction of anthracene with N-methyl-maleinimide.

Photo Diels–Alder reactions in the presence of a sensitizer and light have been described [59–64]. Therefore flavins **1** and **2** were tested as catalyst for the cycloaddition of maleimide to anthracene in toluene (Scheme 5). Table 3 summarizes the results. A significantly higher yield of the cycloaddition product was obtained after 8 h at 40 °C in the presence of compound **2** (entry 3), if compared to the control reaction (entry 6). Upon irradiation with blue light the yield after 8 h reaction time increased further (entry 2) and was significantly higher as in the absence of a photocatalyst (entry 5). However, a comparison with tetraacetyl riboflavin (**3**) under identical reaction conditions showed an even more pronounced acceleration of the reaction (entry 4). Blue light irradiated flavins accelerate the anthracene maleimide cycloaddition significantly, but flavins **1** and **2** do not provide additional benefit if compared to tetraacetyl flavin **3**.

**Table 3:** Results of photoinduced Diels–Alder-reaction.

Entry	Catalyst	hν	Yield (%)	TON	TOF (h^−1^)

1	**1**	+	45	22.5	2.8
2	**2**	+	85	42.5	5.3
3	**2**	–	59	28.5	3.6
4	**3**	+	100	50	6.3
5	–	+	30	**–**	**–**
6	–	–	9	**–**	**–**
7^a^	–	–	100	**–**	**–**

Conditions: Toluene, V = 1.2 mL, anthracene 33 × 10^−3^ M, maleinimide 2.5 equiv, catalyst 2 mol%, t = 8 h, 40 °C, LED (440 nm). ^a^Anthracene 500 μmol, methyl maleinimide 1.25 mmol, toluene 10 mL, 100 °C, 16 h.

## Conclusion

We have prepared new flavin derivatives that bear an acyl guanidinium group, which is linked to the chromophore via a rigid Kemp’s acid spacer. The connectivity and expected relative geometry of **1** and of the carboxylic acids **6** and **9** was confirmed by X-ray structure analysis. Guanidinium cations are known to bind oxoanions, such as phosphates, via hydrogen bonds. Therefore a benefit to the photocatalytic activity of **1** and **2** was expected, as the binding site could keep reaction substrates in close proximity to the redox active chromophore, facilitating photoinduced electron transfer processes. Initial exemplary photocatalytic experiments showed that flavin-derivatives **1** and **2** catalyze oxidative benzyl ether cleavage, nitro arene reductions and Diels–Alder reactions. However, no significant gain in photocatalytic performance by the guanidinium ion substrate binding site was observed in comparison to flavins lacking the binding site and the rigid Kemp’s acid skeleton. The primary interaction between the aromatic substrates and the heteroaromatic flavin chromophore seems to dominate the formation of the substrate–catalyst aggregate. Hydrogen bonds between the substrate and the acylguanidinium group are not decisive for their interaction. The rigidity of the Kemp’s triacid skeleton is not effectively transferred in **1** and **2** to the relative flavin–guanidinium ion orientation, which is due to the flexible ethane linker between imide and flavin. Derivatives with a more constrained conformation of the flavin chromophore and the substrate binding sites may lead to chemical photocatalysts with better performance.

## Experimental

### General

The flavin salts **4** [10-(2-aminoethyl)-3,7,8-trimethylbenzo[g]pteridine-2,4(3*H*,10*H*)-dione] and **8** [3-(2-aminoethyl)-10-(2-methoxyethyl)-7,8-dimethylbenzo[g]pteridine-2,4(3*H*,10*H*)-dione], Kemp’s acid anhydride **5** (1,5,7-trimethyl-2,4-dioxo-3-oxa-bicyclo[3.3.1]nonane-7-carboxylic acid) and mono Boc-protected guanidine were prepared by known methods [21,50–52]. All other chemicals were purchased from commercial suppliers, checked by ^1^H NMR spectrometry and then used as received. Solvents were distilled before use. Flash column chromatography was carried out on silica gel 35–70 μm, 60 Å from Acros. NMR spectra were recorded at a Bruker Avance 300 spectrometer (300 MHz) or at a Bruker Avance 600 spectrometer (600 MHz). Electrospray ionisation (ES-MS) mass spectra were measured on ThermoQuest Finnigan TSQ 7000 spectrometer. High resolution mass spectrometry (HRMS) was measured on ThermoQuest Finnigan MAT 95 spectrometer. Melting points were measured on a Büchi SMP-20 apparatus and are not corrected. IR spectra were measured on Biorad Spectrometer Excalibur FTS 3000. UV/Vis spectra were recorded at Varian Cary 50 Bio UV/VIS spectrometer against air. Fluorescence spectra were recorded at Varian Cary Eclipse.

### 1,5,7-Trimethyl-2,4-dioxo-3-[2-(3,7,8-trimethyl-2,4-dioxo-3,4-dihydrobenzo[*g*]pteridin-10(2*H*)-yl)ethyl]-3-azabicyclo[3.3.1]nonane-7-carboxylic acid; Flavin–Kemp’s acid 6

DMAP (230 mg, 1.9 mmol) and Kemp’s acid anhydride (**5**) (180 mg, 750 μmol) were added successively to a solution of flavin salt **4** (250 mg, 750 μmol) in water (22 mL) and the solution was refluxed for 20 h. After cooling, the mixture was brought to pH 1 with hydrochloric acid (5 M), and the precipitating orange product was collected by filtration. As thin layer chromatography showed considerable amounts of the product in the filtrate, it was concentrated and purified by flash column chromatography (CHCl_3_:MeOH – 15:1), to yield another portion of orange solid. Yield: 302 mg, 580 μmol, 78%, orange solid; *R*_f_ = 0.2 (CHCl_3_:MeOH − 10:1); mp 292 °C (decomp.); ^1^H NMR (DMSO-*d_6_*) δ = 0.87 (s, 6 H, 2 × Kemps-C*H*_3_), 1.00 (s, 3 H, Kemps-C*H*_3_), 1.07–1.26 (m, 3 H, *H*_ax_), 2.20–2.24 (m, 3 H, *H*_eq_), 2.41 (s, 3 H, Ar-C*H*_3_), 2.50 (s, 3 H, Ar-C*H*_3_, hidden by DMSO), 3.28 (s, 3 H, N-C*H*_3_), 3.90 (s, 2 H, C*H*_2_), 4.87 (s, 2 H, C*H*_2_), 7.77 (s, 1 H, Ar-*H*), 7.98 (s, 1 H, Ar-*H*), 12.24 (br s, 1 H, COO*H*); ^13^C NMR (DMSO-*d_6_*) *δ* = 18.8 (Ar-*C*H_3_), 20.9 (Ar-*C*H_3_), 24.7 (2 × *C*H_3_), 28.0 (N-*C*H_3_), 29.7 (*C*H_3_), 37.3 (N-CH_2_), 39.4 (2 × *C_qu_*), 40.9 (2 × *C*H_2_), 41.1 (*C_qu_*), 42.1 (*C*H_2_), 43.0 (N-CH_2_), 116.0(*C*-9), 131.1 (*C*-9a), 131.3 (*C*-6), 134.2 (*C*-5a), 135.5 (*C*-4a), 136.2 (*C*-7), 146.9 (*C*-8), 149.4 (*C*-10a), 154.9 (*C*-2), 159.5 (*C*-4), 176.2 (2 × *C*O), 176.5 (*C*O); ES-MS *m/z* (%): 522.4 (100) [M+H]^+^; HRMS–EI *m/z*: calcd for C_23_H_32_N_5_O_6_ [M+H]^+^: 522.2353; found: 522.2342 [Δ 2.03 ppm]; IR (ATR): ν = 1717, 1649, 1583, 1545, 1451, 1250, 1193, 1096, 1053, 970, 756 cm^−1^.

### 3-{2-[10-(2-Methoxyethyl)-7,8-dimethyl-2,4-dioxobenzo[g]pteridin-3(2*H*,4*H*,10*H*)-yl]ethyl}-1,5,7-trimethyl-2,4-dioxo-3-azabicyclo[3.3.1]nonane-7-carboxylic acid; Flavin–Kemp’s acid 9

DMAP (460 mg, 2.8 mmol) and Kemp’s anhydride (**5**) (370 mg, 1.54 mmol) were added successively to a solution of flavin salt **8** (570 mg, 1.50 mmol) in water (50 mL) and the solution was refluxed for 20 h. After cooling, the mixture was brought to pH 1 with hydrochloric acid (5 M), and the precipitating dark orange product was collected by filtration. As thin layer chromatography showed considerable amounts of the product in the filtrate, it was concentrated and purified by flash column chromatography (CHCl_3_:MeOH – 15:1), to yield another portion of orange solid. Yield: 597 mg, 1.06 mmol, 71%, orange solid; *R*_f_ = 0.2 (CHCl_3_:MeOH – 10:1); mp 305 °C (decomp.); ^1^H NMR (DMSO-*d_6_*) δ = 0.97 (s, 6 H, 2 × Kemps-C*H*_3_), 1.04 (s, 3 H, Kemps-C*H*_3_), 1.15 (d, *J* = 13.72 Hz, 2 H, *H*_ax_), 1.32 (d, *J* = 12.62 Hz, 1 H, *H*_ax_), 1.82 (d, *J* = 12.62 Hz, 1 H, *H*_eq_), 2.25 (d, *J* = 13.17 Hz, 2 H, *H*_eq_), 2.41 (s, 3 H, Ar-C*H*_3_), 2.50 (s, 3 H, Ar-C*H*_3_, hidden by DMSO), 3.22 (s, 3 H, O-C*H*_3_), 3.74–3.77 (m, 4 H, 2 × C*H*_2_), 4.06–4.07 (m, 2 H, N-C*H*_2_), 4.83 (tr, *J* = 5.35 Hz, 2 H, O-C*H_2_*), 7.91 (s, 1 H, Ar-*H*), 7.98 (s, 1 H, Ar-*H*), 12.25 (br s, 1 H, COO*H*); ^13^C NMR (DMSO-*d_6_*) δ = 18.8 (Ar-*C*H_3_), 20.8 (Ar-*C*H_3_), 25.0 (*C*H_3_), 29.9 (*C*H_3_), 37.4, 41.0, 41.6 and 43.1 (*C_qu_* and *C*H_2_), 44.0 (10-N-*C*H_2_), 58.5 (O-*C*H_3_), 68.3 (O-*C*H_2_), 116.9 (*C9*), 130.9 (*C6*), 131.5 (*C9a*), 134.0 (*C5a*), 135.6 (*C4a*), 136.3 (*C7*), 147.0 (*C8*), 148.6 (*C10a*), 155.0 (*C2*), 159.7 (*C4*), 176.1 und 176.6 (*C*OOH und *C*ONH); ES-MS *m/z* (%): 566.3 (100) [M+H]^+^; HRMS–EI *m/z*: calcd for C_29_H_36_N_5_O_7_ [M+H]^+^: 566.2615; found: 566.2623 [Δ −1.46 ppm]; IR (ATR): ν = 1719, 1673, 1621, 1581, 1548, 1234, 1119, 953, 886, 806, 758 cm^−1^.

### N-(Boc-Carbamimidoyl)-1,5,7-trimethyl-2,4-dioxo-3-{2-[3,7,8-trimethyl-2,4-dioxo-3,4-dihydrobenzo[g]pteridin-10(2*H*)-yl]ethyl}-3-azabicyclo[3.3.1]nonane-7-carboxamide; Flavin-Boc-guanidin 7

To a solution of HOBt·H_2_O (89 mg, 580 μmol), EDC (90 mg, 580 μmol) and DIPEA (171 μL, 970 μmol) in CH_2_Cl_2_ (6.5 mL) were added compound **6** (252 mg, 480 μmol) and mono Boc-protected guanidine (86 mg, 530 μmol) at 0 °C. The mixture was stirred at room temperature for 20 h, diluted with CHCl_3_ (150 mL) and washed with brine twice. The organic phase was separated, dried over magnesium sulfate and the solvents were evaporated to yield an orange solid. The crude product was purified by flash column chromatography (CHCl_3_:MeOH – 50:1). Yield: 186 mg, 280 μmol, 58%, orange solid; *R*_f_ = 0.15 (CHCl_3_:MeOH – 50:1); mp 255–259 °C (decomp.); ^1^H NMR (CDCl_3_) δ = 0.91–1.05 (m, 12 H, 3 × C*H_3_* + 3 × H*_ax_*), 1.48 (s, 9 H, Boc-C*H_3_*), 2.39 (s, 3 H, Ar-C*H_3_*), 2.48 (s, 3 H, Ar-C*H_3_*), 2.64–2.69 (m, 3 H, *H_eq_*), 3.48 (s, 3 H, N-C*H_3_*), 3.98 (tr, *J* = 4.53 Hz, 2 H, C*H_2_*), 4.84 (br s, 2 H, C*H_2_*), 7.30 (s, 1 H, Ar-*H*) 7.99 (s, 1 H, Ar-*H*), 8.33 (br s, 1 H, N-*H*), 8.85 (br s, 1 H, N-*H*); ^13^C NMR (CDCl_3_) δ = 19.5 (Ar-*C*H_3_) 21.9 (Ar-*C*H_3_), 25.5 (2 × *C*H_3_), 28.1 (Boc-*C*H_3_), 28.8 (N-*C*H_3_), 31.3 (*C*H_3_), 37.2 (N-*C*H_2_), 40.1 (2 × *C_qu_*), 42.3 (*C*H_2_), 43.3 (*C*H_2_), 44.1 (N-*C*H_2_), 44.5 (*C_qu_*), 83.8 (Boc-*C_qu_*), 115.1 (*C9*), 131.9 (*C9a*), 132.8 (*C6*), 134.7 (*C5a*), 135.8 (*C4a*), 136.3 (*C7*), 146.8 (*C8*), 149.3 (*C10a*), 153.0 (NH*C*O), 156.0 (*C2*), 158.7 (Boc-*C*O), 160.2 (*C4*), 177.5 (NH*C*O); ES-MS *m/z* (%): 681.4 [M+NH_4_]^+^, 663.4 (100) [M+H]^+^, 563.3 [M+H-Boc]^+^; HRMS–EI *m/z*: calcd for C_33_H_43_N_8_O_7_ [M+H]^+^: 663.3255; found: 663.3242 [Δ 1.92 ppm] IR (ATR): ν = 1716, 1668, 1634, 1584, 1543, 1455, 1367, 1327, 1236, 1144, 968, 756 cm^−1^.

### N-(Boc-Carbamimidoyl)-3-{2-[10-(2-methoxyethyl)-7,8-dimethyl-2,4-dioxobenzo[g]pteridin-3(2*H*,4*H*,10*H*)-yl]ethyl}-1,5,7-trimethyl-2,4-dioxo-3-azabicyclo[3.3.1]nonane-7-carboxamide; Flavin-Boc-guanidin 10

To a solution of HOBt·H_2_O (226 mg, 1.67 mmol), EDC (226 mg, 1.45 mmol) and DIPEA (498 μL, 970 μmol) in CHCl_3_ (10 mL) was added compound **9** (548 mg, 969 μmol) and mono Boc-protected guanidine (231 mg, 1.45 mmol) at 0 °C. The mixture was stirred at room temperature for 20 h, diluted with CHCl_3_ (250 mL) and washed with water and brine. The organic phase was separated, dried over magnesium sulfate and the solvents were evaporated. The crude brown product was purified by flash column chromatography (CHCl_3_:MeOH:N(Et)_3_ – 70:1:1). Yield: 564 mg, 798 μmol, 82%, yellow solid; *R*_f_ = 0.1 (CHCl_3_:MeOH:TEA – 50:1:1); mp 229–231 °C (decomp.); ^1^H NMR (CDCl_3_) δ = 0.93–1.21 (m, 12 H, 3 × C*H_3_* + 3 × H*_ax_*), 1.46 (s, 9 H, Boc-C*H_3_*), 2.15 (d, *J* = 12.90 Hz, 1 H, *H_eq_*), 2.38 (s, 3 H, Ar-C*H_3_*), 2.48 (s, 3 H, Ar-C*H_3_*), 2.68 (d, *J* = 13.44 Hz, 2 H, *H_eq_*), 3.22 (s, 3 H, O-C*H_3_*), 3.80–3.84 (m, 4 H, 2 × C*H_2_*), 4.20–4.22 (m, 2 H, C*H_2_*), 4.79 (tr, *J* = 5.08 Hz, 2 H, C*H_2_*), 7.58 (s, 1 H, Ar-*H*) 7.94 (s, 1 H, Ar-*H*), 8.27 (br s, 1 H, N-*H*), 8.75 (br s, 1 H, N-*H*); ^13^C NMR (CDCl_3_) δ = 19.5 (Ar-*C*H_3_) 21.5 (Ar-*C*H_3_), 25.5 (2 × *C*H_3_), 28.1 (Boc-*C*H_3_), 31.3 (*C*H_3_), 38.4 (N-*C*H_2_), 40.2 (2 × *C_qu_*), 40.8 (N-*C*H_2_), 43.2 (N-*C*H_2_), 44.2 (2 × *C*H_2_), 44.6 (*C_qu_*), 45.2 (*C*H_2_), 59.2 (O-*C*H_3_), 69.6 (O-*C*H_2_), 83.4 (Boc-*C_qu_*), 116.6 (*C9*), 132.2 (*C9a*), 132.2 (*C6*), 134.9 (*C5a*), 135.6 (*C4a*), 136.4 (*C7*), 147.2 (*C8*), 148.7 (*C10a*), 153.4 (NH*C*O), 156.1 (*C2*), 158.5 (Boc-*C*O), 160.4 (*C4*), 177.5 (NH*C*O), 188.7 (*C_qu_*); ES-MS *m/z* (%): 707.3 (100) [M+H]^+^; HRMS–EI *m/z*: calcd for C_35_H_47_N_8_O_8_ [M+H]^+^: 707.3517; found: 707.3531 [Δ −2.00 ppm]; IR (ATR): ν = 1706, 1655, 1634, 1583, 1540, 1457, 1366, 1325, 1226, 1146, 758 cm^−1^.

### N-Carbamimidoyl-1,5,7-trimethyl-2,4-dioxo-3-{2-[3,7,8-trimethyl-2,4-dioxo-3,4-dihydrobenzo[g]pteridin-10(2*H*)-yl]ethyl}-3-azabicyclo[3.3.1]nonane-7-carboxamide; Flavin-guanidinium 1

Compound **7** (210 mg, 317 μmol) was dissolved in CHCl_3_ (25 mL) and hydrogen chloride saturated diethyl ether (3 mL) was added dropwise. After stirring for 24 h, the solution was evaporated to 5 mL and diethyl ether (15 mL) was added to precipitate the product. The mixture was cooled to 0 °C and the solid was filtered off, washed with diethyl ether and dried. Yield: 157 mg, 262 μmol, 83%, orange-yellow solid; *R*_f_ = 0.1 (CHCl_3_:MeOH – 10:1); mp 320–322 °C (decomp.); ^1^H NMR (DMSO-*d_6_*) δ = 0.91 (s, 6 H, 2 × C*H_3_*), 1.16 (s, 3 H, C*H_3_*), 1.28–1.31 (m, 4 H, 3 × C*H_ax_* and C*H_eq_*), 2.41 (Ar-C*H_3_*), 2.47 (d, *J* = 14.39 Hz, 2 H, *H_eq_*), 2.50 (Ar-C*H_3_*, hidden by DMSO), 3.28 (s, 3 H, N-C*H_3_*), 3.90 (tr, *J* = 5.01 Hz, 2 H, C*H_2_*), 4.84 (s, 2 H, C*H_2_*), 7.74 (Ar-*H*), 7.97 (Ar-*H*), 8.36–8.44 (m, 4 H, N*H*), 11.38 (s, 1 H, N*H*CO); ^13^C NMR (DMSO-*d_6_*) δ = 18.7 (Ar-*C*H_3_) 21.0 (Ar-*C*H_3_), 24.7 (2 × *C*H_3_), 28.0 (N-*C*H_3_), 29.0 (*C*H_3_), 36.8 (*C*H_2_), 40.1 (*C_qu_*), 40.7 (*C*H_2_), 42.0 (*C*H_2_+*C_qu_*), 43.6 (*C*H_2_), 116.0 (*C9*), 131.1 (*C5a*), 131.3 (*C6*), 134.0 (*C9a*), 135.5 (*C4a*), 136.2 (*C7*), 146.8 (*C8*), 149.3 (*C10a*), 154.9 (*C2*), 155.0 (*C_qu_*), 159.4 (*C4*), 176.0 (*C*O), 177.3 (*C*O); ES-MS *m/z* (%): 563.3 (100) [M+H]^+^; HRMS–EI *m/z*: calcd for C_28_H_35_N_8_O_5_ [M+H]^+^: 563.2730; found: 563.2746 [Δ −2.77 ppm]; IR (ATR): ν = 1700, 1643, 1584, 1546, 1452, 1306, 1238, 1190, 1127, 1098, 1048, 753 cm^−1^; UV/Vis (MeCN): λ_max_ (ε) = 272 (41500), 343 (9130), 447 nm (11060); Fluorescence (MeCN): λ_max_ (emission) = 507 nm (excitation: 445 nm).

### N-Carbamimidoyl-3-{2-[10-(2-methoxyethyl)-7,8-dimethyl-2,4-dioxobenzo[g]pteridin-3(2*H*,4*H*,10*H*)-yl]ethyl}-1,5,7-trimethyl-2,4-dioxo-3-azabicyclo[3.3.1]nonane-7-carboxamide; Flavin-guanidinium 2

Compound **10** (493 mg, 698 μmol) was dissolved in CHCl_3_ (50 mL) and hydrogen chloride saturated diethyl ether (6 mL) was added dropwise. After stirring for 24 h, the solution was evaporated to 5 mL and diethyl ether (25 mL) was added to precipitate the product. The mixture was cooled to 0 °C and the solid was filtered off, washed with diethyl ether and dried. Yield: 402 mg, 625 μmol, 90%, yellow solid; *R*_f_ = 0.15 (CHCl_3_:MeOH – 10:1); mp 245–247 °C (decomp.); ^1^H NMR (DMSO-*d_6_*) δ = 0.99 (s, 6 H, 2 × C*H_3_*), 1.17 (s, 3 H, C*H_3_*), 1.30 (d, *J* = 14.39 Hz, 2 H, 2 × C*H_ax_*), 1.38 (d, *J* = 12.59 Hz, 1 H, C*H_ax_*), 1.84 (d, *J* = 12.59 Hz, H, C*H_eq_*), 2.39 (s, 3 H, Ar-C*H_3_*), 2.51 (s, 3 H, Ar-C*H_3_*), 2.52 (d, *J* = 14.39 Hz, 2 H, 2 × CH_ax_), 3.21 (s, 3 H, O-C*H_3_*), 3.73–3.75 (m, 4 H, 2 × C*H_2_*), 4.02–4.04 (m, 2 H, C*H_2_*), 4.82 (tr, *J* = 5.52 Hz, 2 H, C*H_2_*), 7.89 (Ar-*H*), 7.95 (Ar-*H*), 8.34–8.43 (m, 4 H, N*H*), 11.42 (s, 1 H, N*H*CO); ^13^C NMR (DMSO-*d_6_*) δ = 18.8 (Ar-*C*H_3_) 20.7 (Ar-*C*H_3_), 24.9 (2 × *C*H_3_), 28.6 (*C*H_3_), 37.3 (*C*H_2_), 39.5 (*C*H_2_), 40.1 (*C_qu_*), 41.2 (*C*H_2_), 42.1 (2 × *C*H_2_), 43.8 (*C_qu_*), 44.0 (*C*H_2_), 116.9 (*C9*), 130.9 (*C6*), 131.4 (*C5a*), 134.1 (*C9a*), 135.5 (*C4a*), 136.3 (*C7*), 147.0 (*C8*), 148.5 (*C10a*), 154.9 (*C2*), 155.1 (*C_qu_*), 159.7 (*C4*), 176.0 (*C*O), 177.3 (*C*O); ES-MS *m/z* (%): 607.3 (100) [M+H]^+^; HRMS–EI *m/z*: calcd for C_30_H_39_N_8_O_6_ [M]^+^: 607.2993; found: 607.2981 [Δ 1.90 ppm]; IR (ATR): ν = 1692, 1669, 1579, 1545, 1456, 1328, 1235, 1173, 747 cm^−1^; UV/Vis (MeCN): λ_max_ (ε) = 275 (96000), 344 (8760), 445 nm (10510); Fluorescence (MeCN): λ_max_ (emission) = 509 nm (excitation: 445 nm).

## Supporting Information

Photocatalytic experiments, UV/Vis and fluorescence spectra of **1**–**3**, calculated gas phase conformations, ^1^H and ^13^C NMR spectra of **1**, **2**, **6**, **7**, **9**, and **10**.

File 1 Koenig_Supporting Information

File 2 CIF file of compound **1**.

File 3 CIF file of compound **6**.

File 4 CIF file of compound **9**.
