# Supplementary material for: Synthesis of rigidified flavin–guanidinium ion conjugates and investigation of their photocatalytic properties
Source: Beilstein J Org Chem. 2009 May 28;5:26. doi: 10.3762/bjoc.5.26 (PMC2707025; doi:10.3762/bjoc.5.26)

Synthesis of rigidified flavin–guanidinium ion conjugates and investigation of their photocatalytic properties

Harald Schmaderer, Mouchumi Bhuyan and Burkhard König*

Address: Institute of Organic Chemisty, University of Regensburg, Universitätsstr. 31, D-93040 Regensburg, Germany

Email: Burkhard König - burkhard.koenig@chemie.uni-regensburg.de

* Corresponding author

Supporting Information

Detailed experimental setups for the photocatalytic experiments 2

Calculated gas phase conformations of compound **1** 3

UV/Vis and fluorescence spectra of **1**, **2** and **3** 4

UV- and emission titration of compound **2** and phosphate esters 5

^{1}H and ^{13}C NMR spectra of **6** 8

^{1}H and ^{13}C NMR spectra of **9** 9

^{1}H and ^{13}C NMR spectra of **7** 10

^{1}H and ^{13}C NMR spectra of **10** 11

^{1}H and ^{13}C NMR spectra of **1** 12

^{1}H and ^{13}C NMR spectra of **2** 13

Detailed experimental setups for the photocatalytic experiments

Oxidative photocleavage of dibenzyl phosphate

The photocleavage of dibenzyl phosphate was tested in small glass vials with deuterated solvents (1 mL, MeCN-d_3 or D_2O), containing the phosphate starting material (10×10^{-3} M) and flavin catalyst (2×10^{-3} M, 20 mol%). The mixture was stirred and irradiated by a LED (440 nm, 5 W) at 40 °C for 2 h (D_2O) or 4 h (MeCN-d_3), respectively. The products were identified by ^{1}H NMR and mass spectrometry and the conversion was determined by integration of the aromatic protons in the ^{1}H NMR.

Photoreduction of 4-nitrophenyl phosphate

The photocleavage of dibenzyl phosphate was tested in small glass vials in MeCN or water (5 mL), containing the 4-nitrophenyl phosphate starting material (10×10^{-3} M), triethanol amine as sacrificial electron donor (100×10^{-3} M, 10 equivalents), and flavin catalyst (1×10^{-3} M, 10 mol%). The solution was degassed by three consecutive pump and freeze circles and afterwards, the solution was stirred and irradiated by a LED (440 nm, 5 W) and a UV-lamp (370 nm) at 40 °C for 4 h. Reaction mixtures in MeCN were evaporated and dried. The mixtures of the experiments in water were lyophilized. 4-aminophenyl phosphate and 4-amino phenol as by-product were identified by ^{1}H NMR and mass spectrometry, respectively. The conversion was determined by integration of the aromatic protons in the ^{1}H NMR.

Photo Diels–Alder-reaction of anthracene with N-methyl-maleinimide

Photoinduced Diels–Alder-reactions were carried out in small glass vials in dry toluene (1.2 mL), containing anthracene (33×10^{-3} M), methyl maleinimide (83×10^{-3} M, 2.5 equivalents), and flavin catalyst (0.67×10^{-3} M, 2 mol%). The mixture was stirred and irradiated by a LED (440 nm, 5 W) at 40 °C for 8 h. Afterwards, the solvent was evaporated and the mixture was dried. The products were identified by ^{1}H NMR and mass spectrometry and the conversion was determined by integration of the aromatic protons in the ^{1}H NMR.

Calculated gas phase conformations

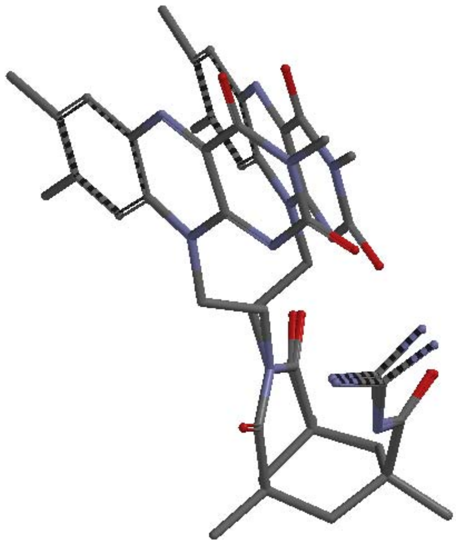

Figure S.1: Calculated conformations of **1** in the gas phase (AM1, Spartan program package).

Figure S.1 shows the two lowest energy conformations of compound **1** in the gas phase (semi-empirical AM1, Spartan program package, difference: 13.5 kJ/mol). It is possible to transform the structures into each other by rotation of the C–C single bonds of the ethane linker that are expected to rotate freely in solution.

UV/Vis and fluorescence spectra of compounds 1, 2 and 3

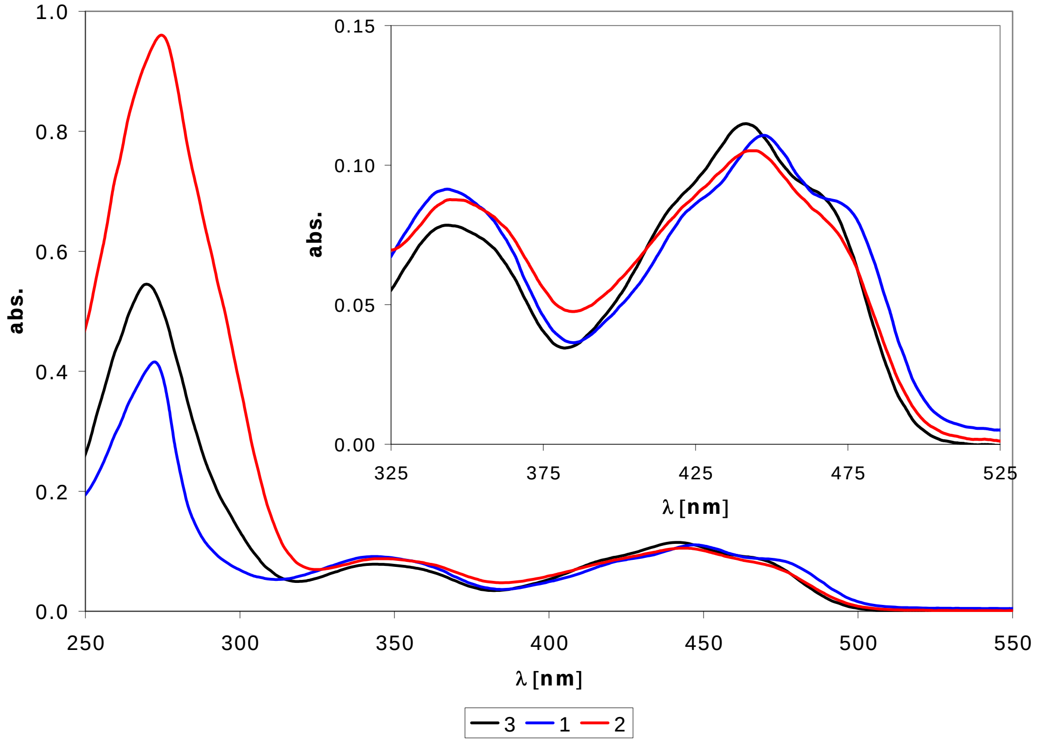

Figure S.2: Absorption spectra of **1**, **2** and **3** (MeCN + 1% DMSO, 1 × 10^{-5} M)

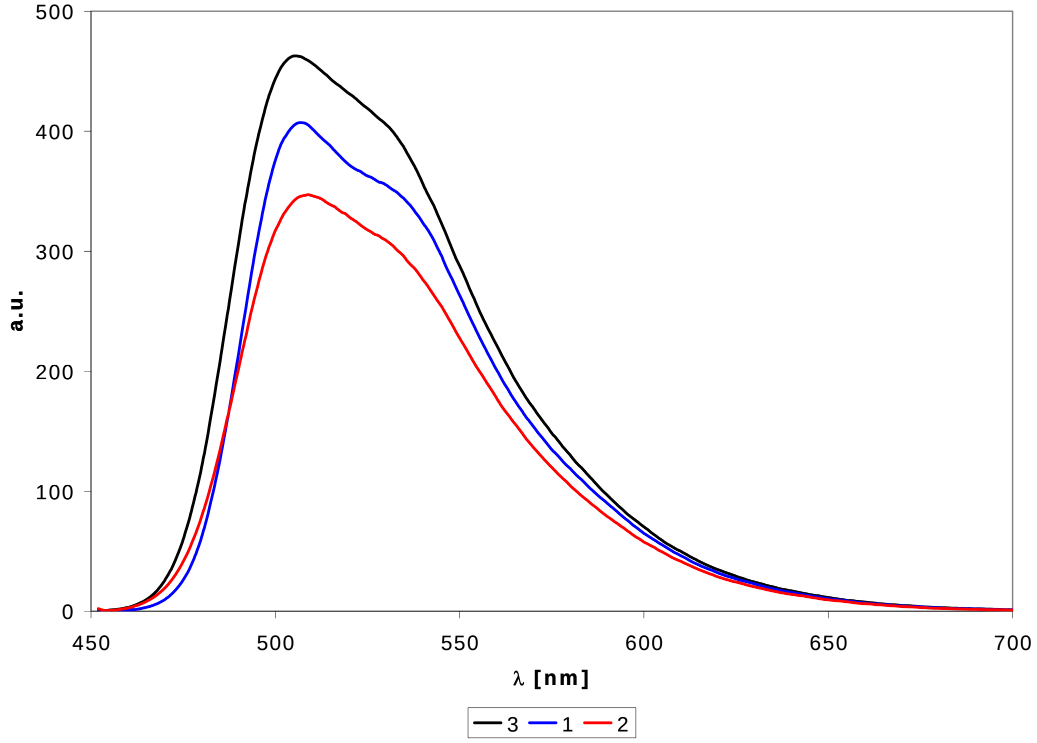

Figure S.3: Fluorescence spectra of **1**, **2** and **3** [MeCN + 1% DMSO, 1 × 10^{-5} M, excitation at 445 nm (**1+2**) and 440 nm (**3**)]

UV- and emission titration of compound 2 and phosphate esters

UV-visible and emission titrations were recorded for compound **2** with sodium phenyl phosphate (dibasic dihydrate) and bis-(4-nitrophenyl) phosphate. UV–visible absorption spectroscopy was performed using a Cary 50 Bio spectrophotometer and fluorescence spectroscopy was performed using a Varian Cary Eclipse fluorescence spectrophotometer. The titration experiments with the phosphate anion species were carried out using a solution (2.5 mL) of compound **2** (50 μM in HEPES Buffer; pH 7.4 and 50 μM in CH_3CN) in a quartz cell at 25 °C. The absorption and emission spectral changes were monitored upon addition of a freshly prepared solution of the analytes (in HEPES Buffer and in CH_3CN) with a microsyringe.

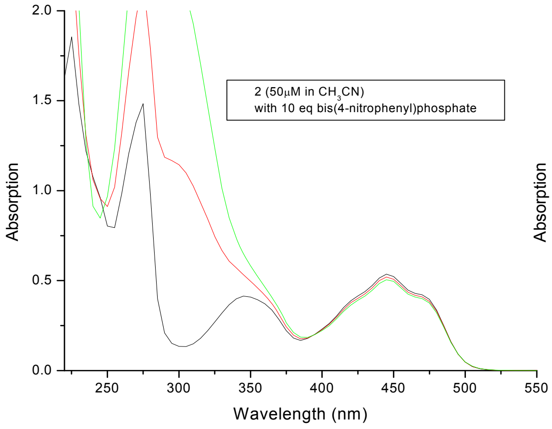

(a)

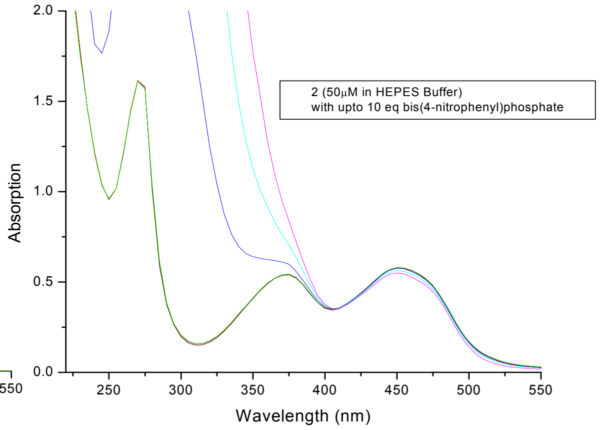

(b)

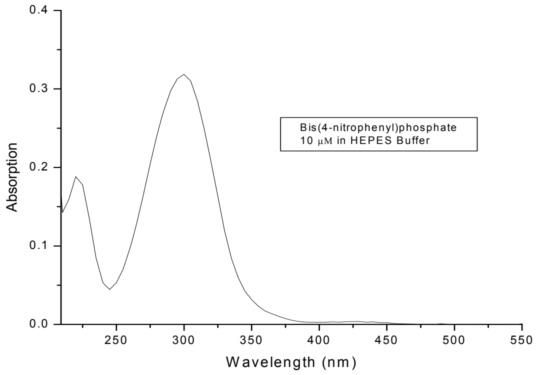

(c)

Figure S.4: UV-Visible spectra of compound **2** (a) with bis (4-nitrophenyl) phosphate in CH_3CN, (b) with bis (4-nitrophenyl) phosphate in HEPES Buffer (c) UV-Visible of bis (4-nitrophenyl) phosphate in HEPES Buffer.

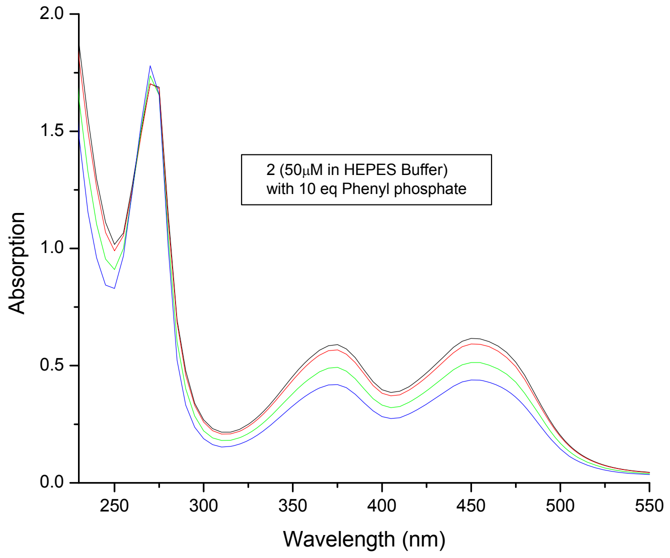

Figure S.5: UV-Visible spectra of compound **2** and sodium phenyl phosphate (dibasic dihydrate) in HEPES Buffer.

The fluorescence spectroscopic studies of compound **2** are shown below. In the presence of bis-(4-nitrophenyl) phosphate as analyte in acetonitrile the emission intensity decreases, while the change is not prominent in HEPES Buffer solution.

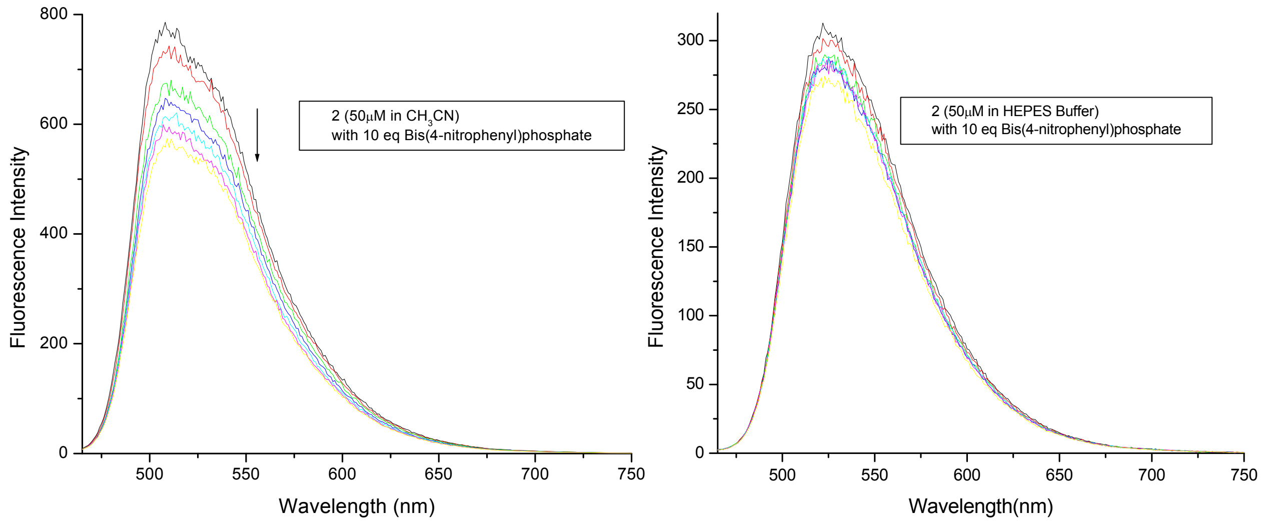

Figure S.6: Fluorescence intensity change of compound **2** with bis (4-nitrophenyl) phosphate in CH_3CN and in HEPES Buffer. λ_{ex} = 445 nm, PMT detector voltage is medium, slit width = 5.

With sodium phenyl phosphate (dibasic dihydrate) as the analyte, compound **2** did not show any significant change in its emission intensity.

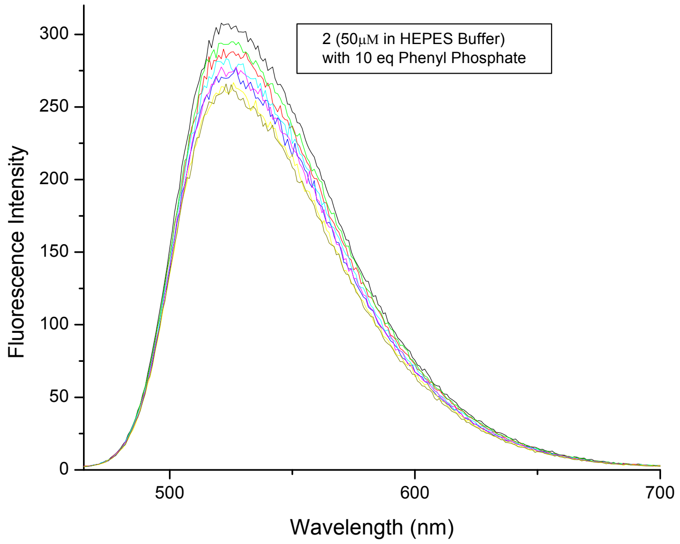

Figure S.7: Fluorescence intensity change of compound **2** with sodium phenyl phosphate (dibasic dihydrate) in HEPES Buffer. λ_{ex} = 445 nm, PMT detector voltage is medium, slit width = 5.

^{1}H NMR spectrum (300 MHz, DMSO-d_6) (top) and ^{13}C NMR (75 MHz, DMSO-d_6) of compound **6**.

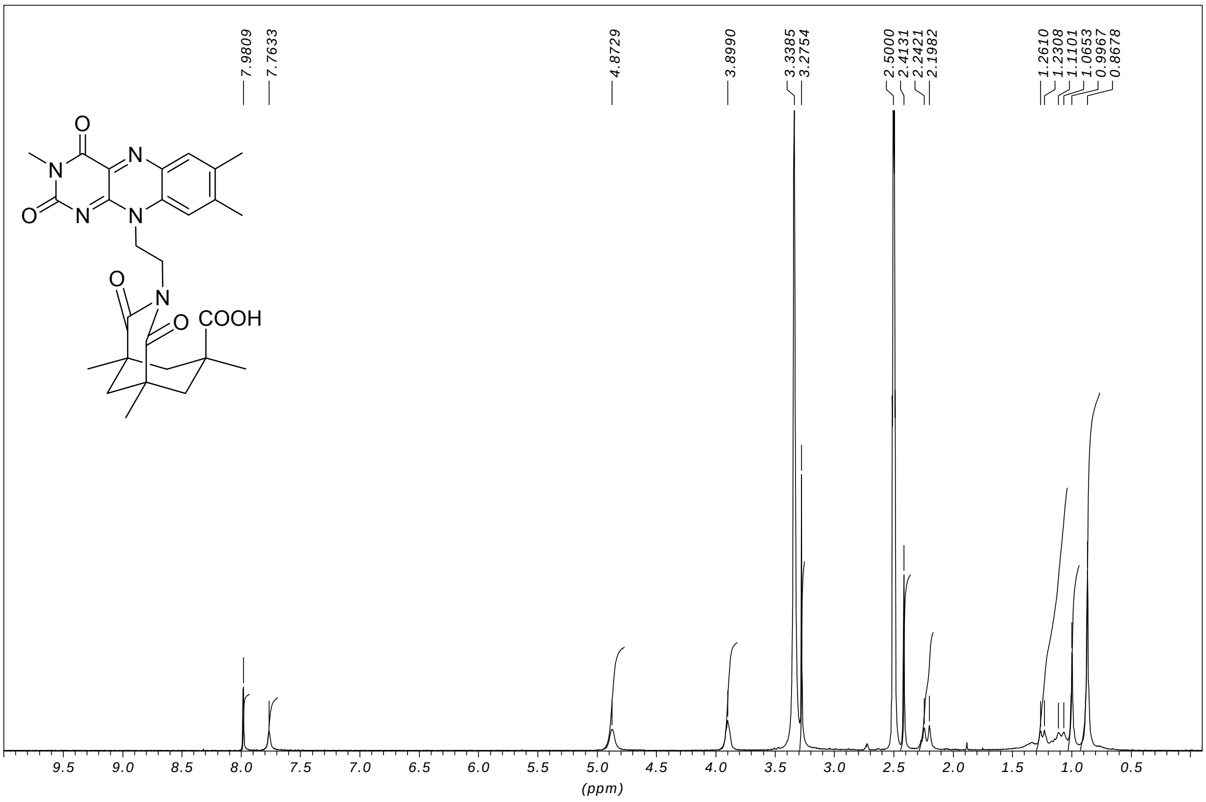

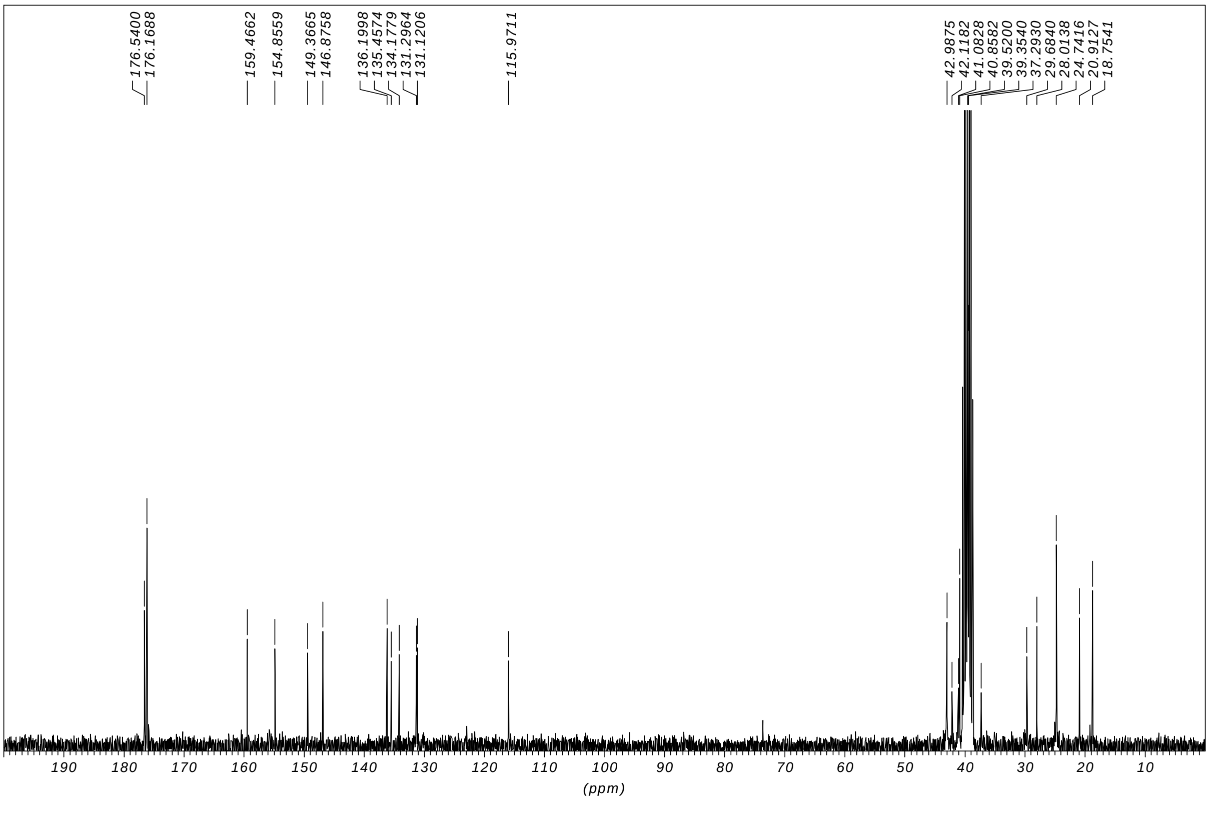

^{1}H NMR spectrum (300 MHz, DMSO-d_6) (top) and ^{13}C NMR (75 MHz, DMSO-d_6) of compound **9**.

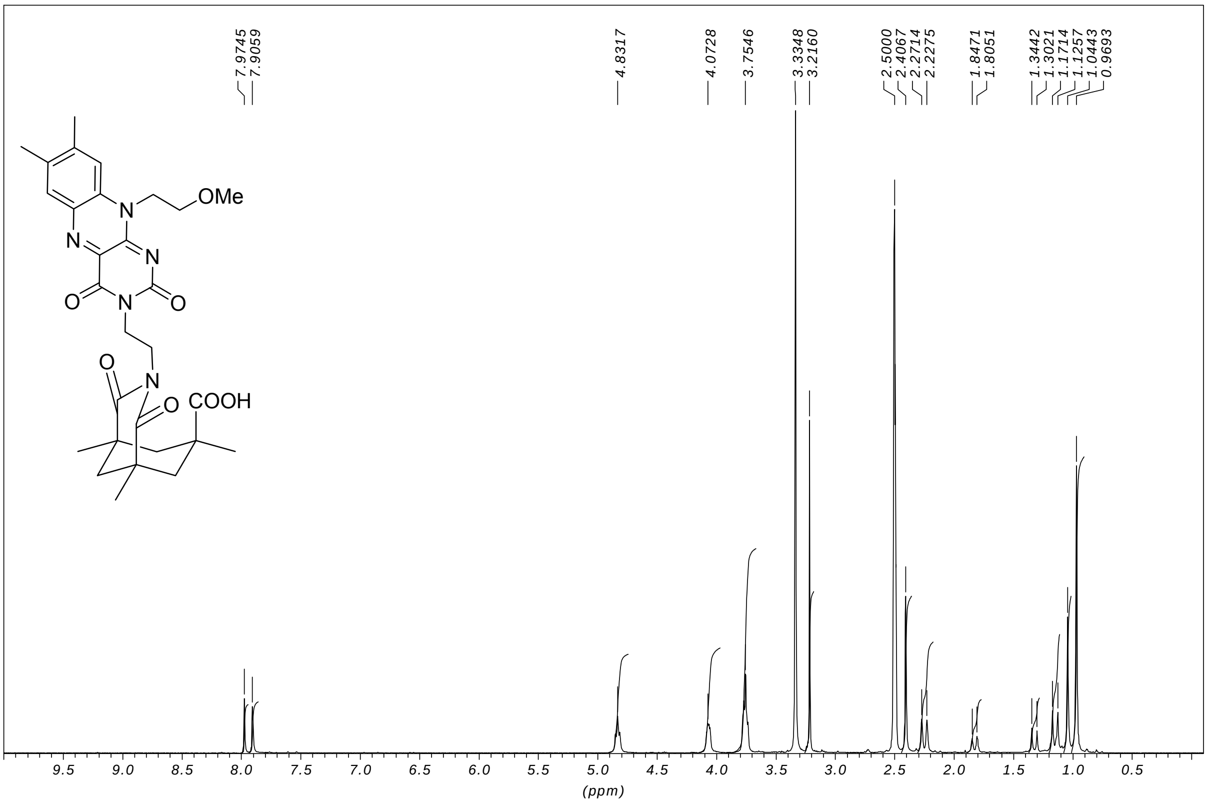

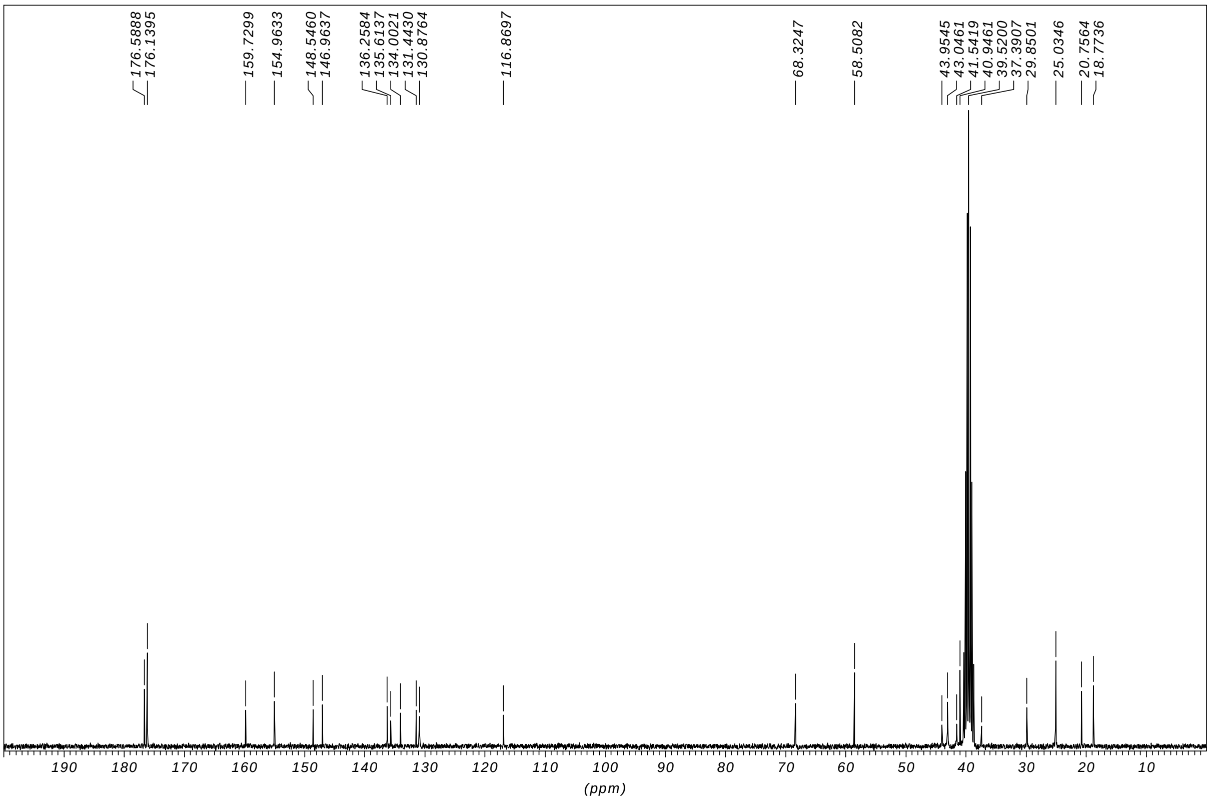

^{1}H NMR spectrum (300 MHz, $CDCl_3$) (top) and ^{13}C NMR (75 MHz, $CDCl_3$) of compound **7**.

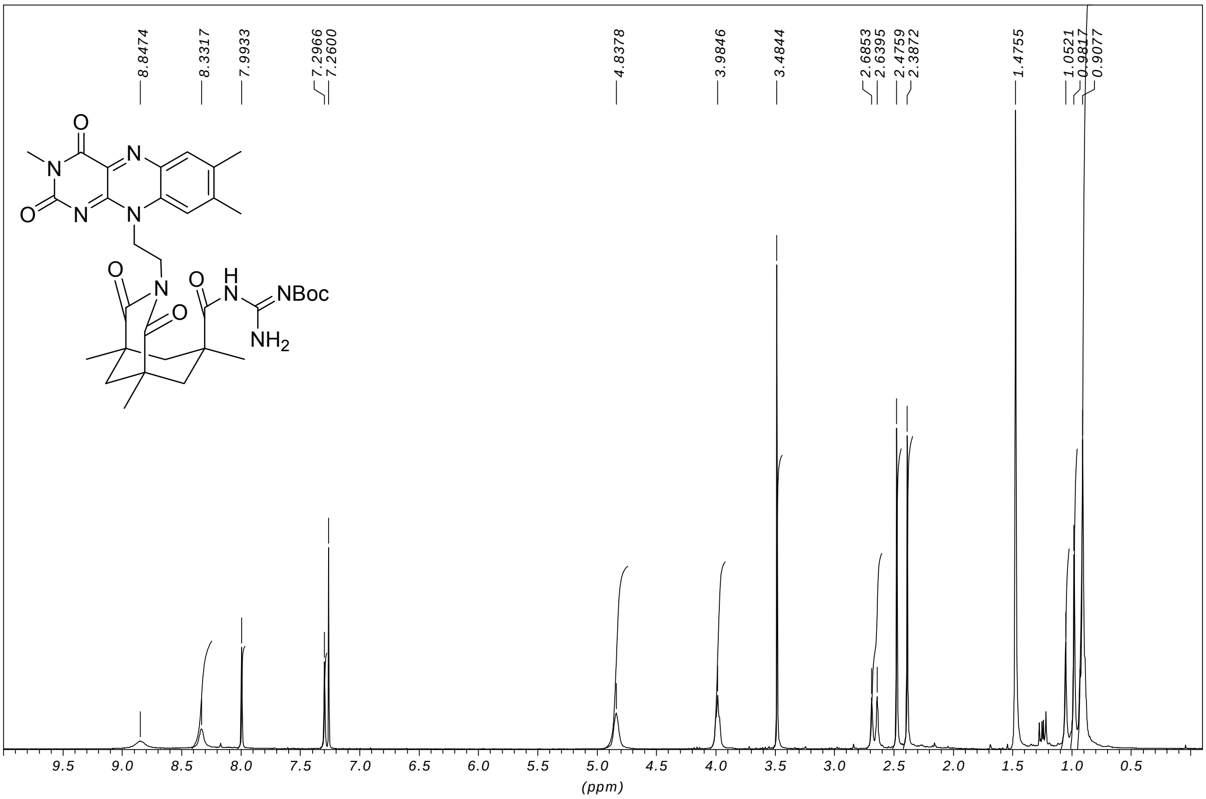

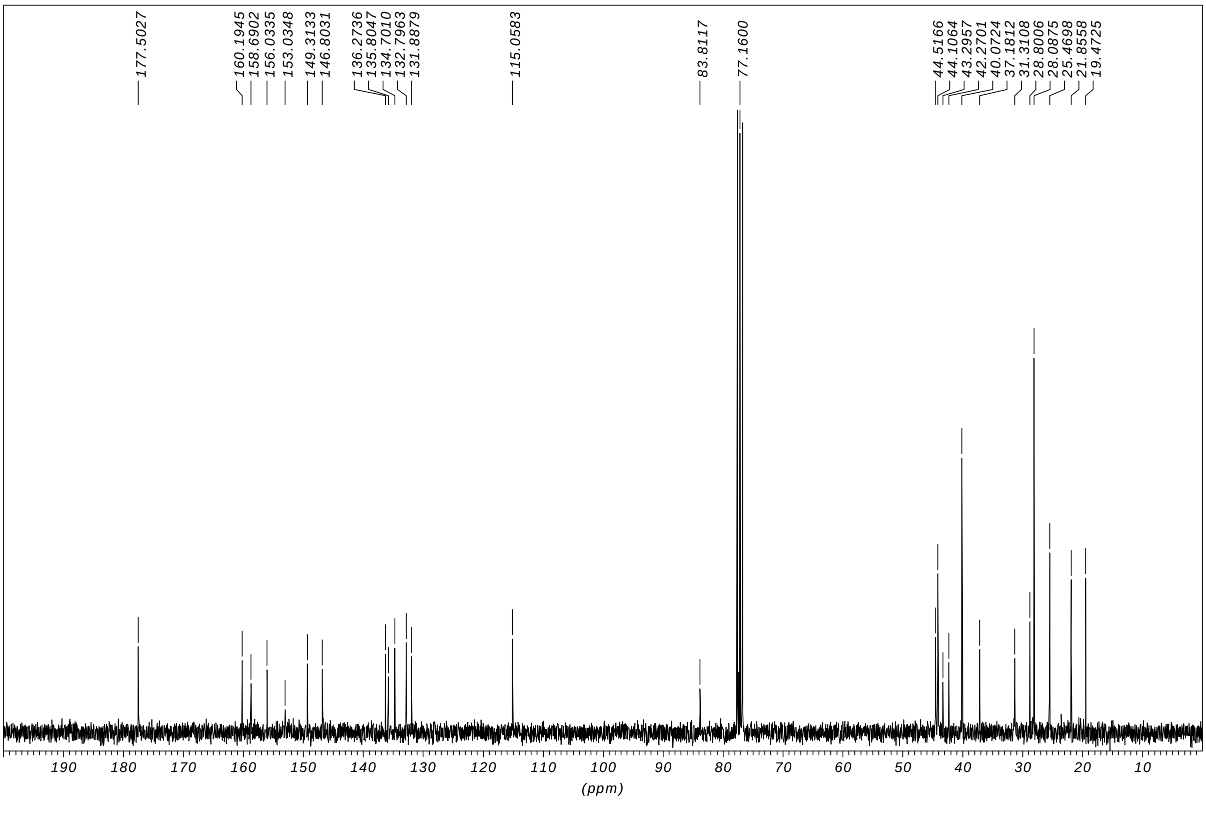

^1H NMR spectrum (300 MHz, $CDCl_3$) (top) and ^{13}C NMR (75 MHz, $CDCl_3$) of compound **10**.

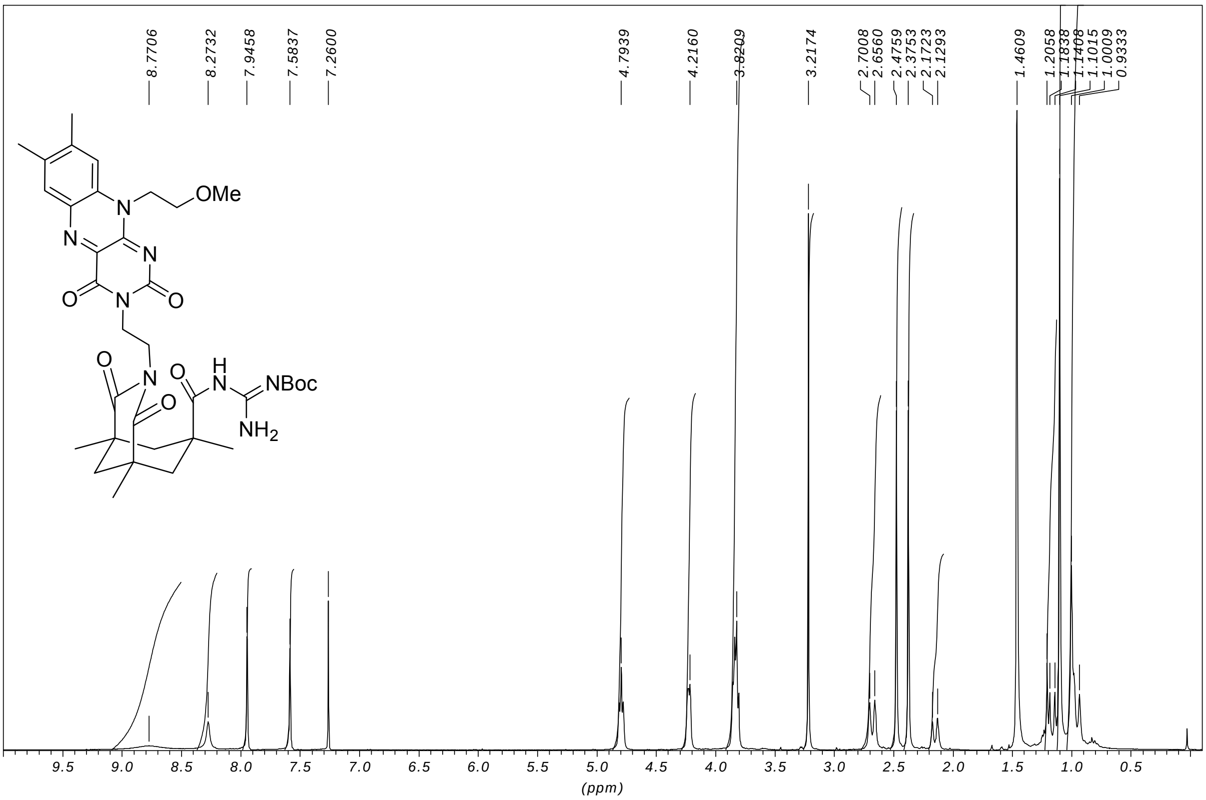

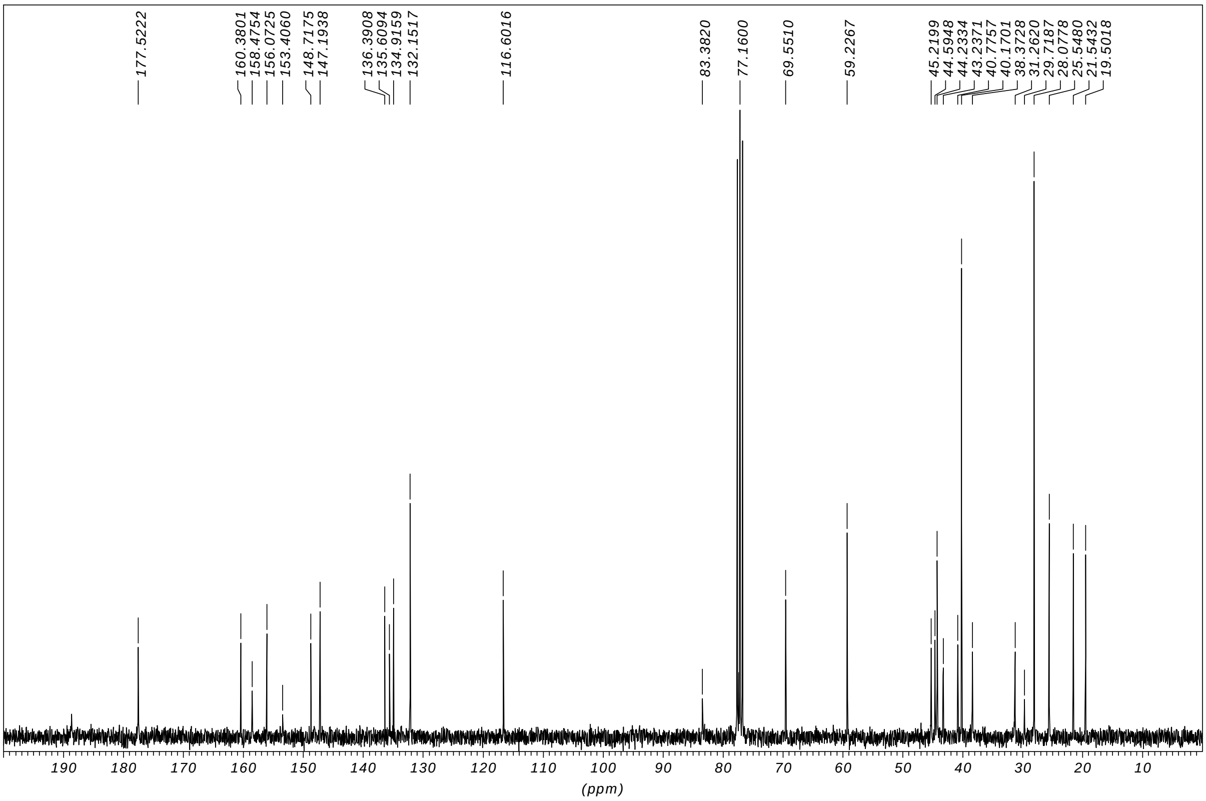

^{1}H NMR spectrum (600 MHz, DMSO-d_6) (top) and ^{13}C NMR (150 MHz, DMSO-d_6) of compound **1**.

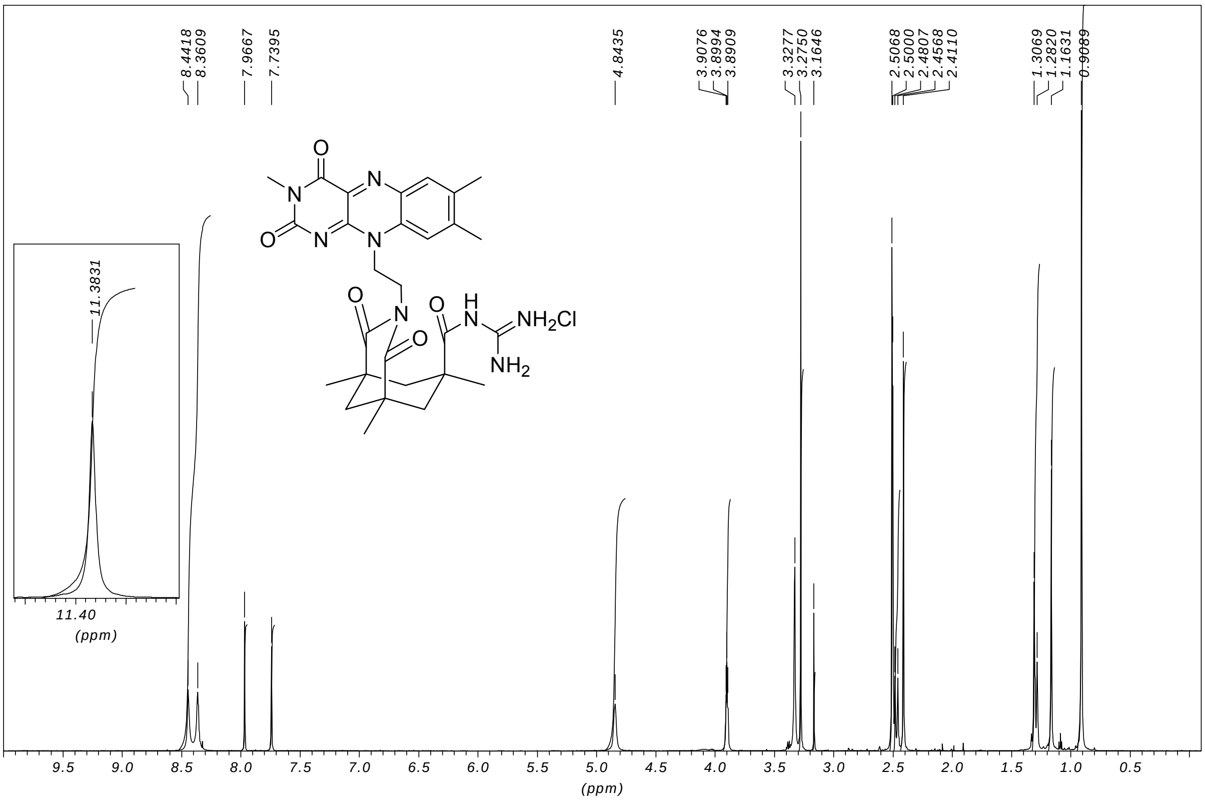

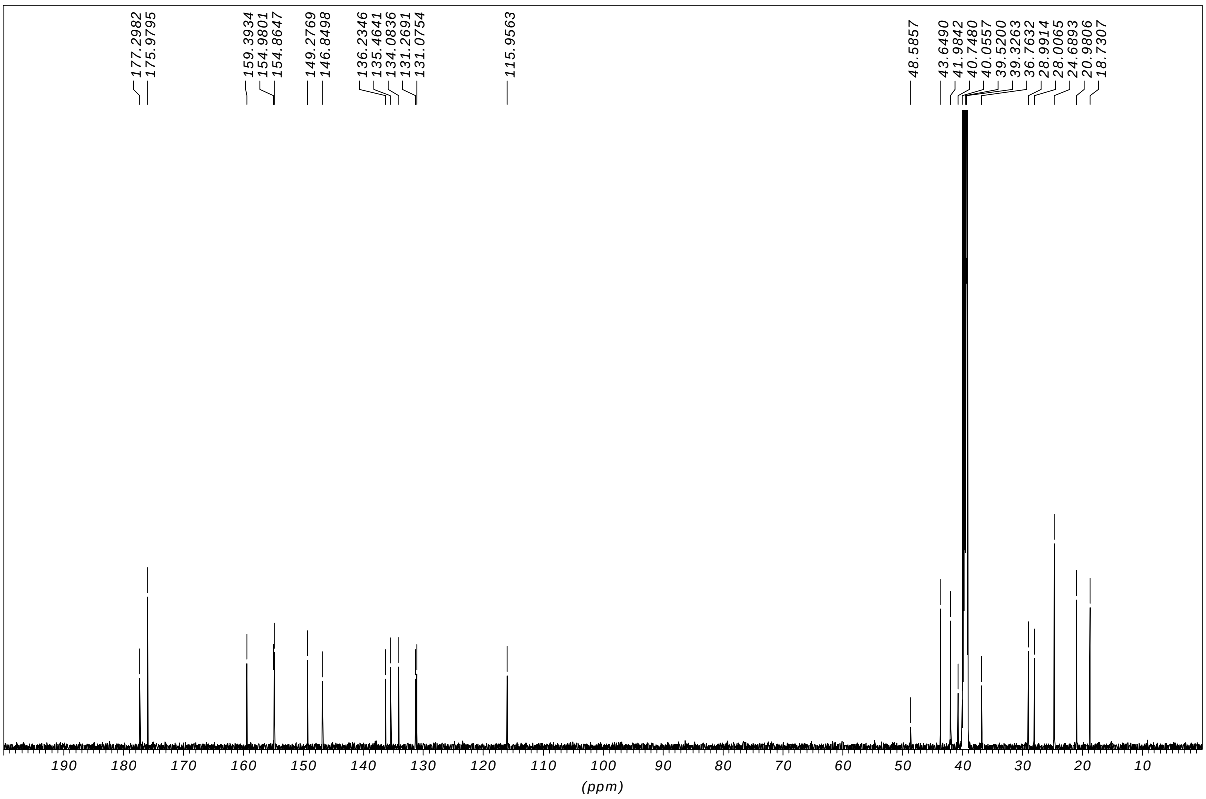

^{1}H NMR spectrum (600 MHz, DMSO-d_6) (top) and ^{13}C NMR (150 MHz, DMSO-d_6) of compound **2**.

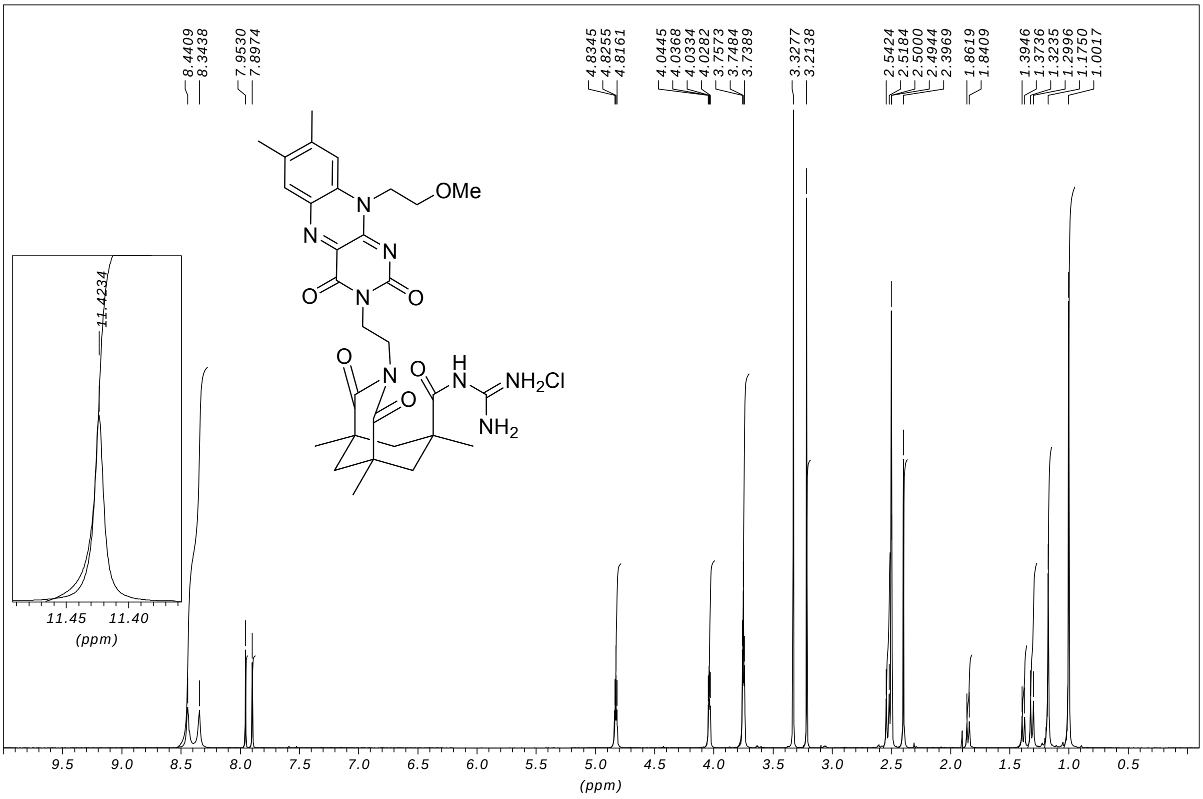

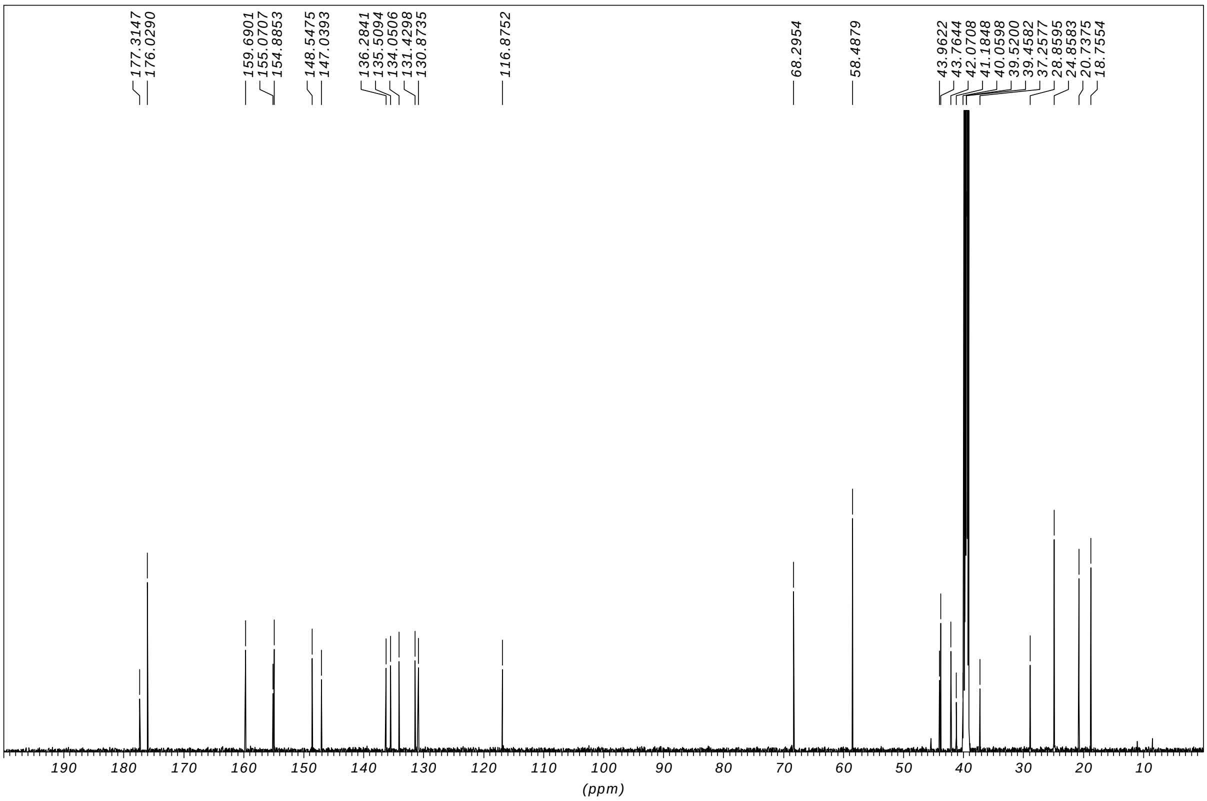

Supplement: File 1 — Koenig_Supporting Information [file Beilstein_J_Org_Chem-05-26-s001.pdf]
